# Differences in Longevity and Temperature-Driven Extrinsic Incubation Period Correlate with Varying Dengue Risk in the Arizona–Sonora Desert Region

**DOI:** 10.3390/v15040851

**Published:** 2023-03-26

**Authors:** Kacey C. Ernst, Kathleen R. Walker, A Lucia Castro-Luque, Chris Schmidt, Teresa K. Joy, Maureen Brophy, Pablo Reyes-Castro, Rolando Enrique Díaz-Caravantes, Veronica Ortiz Encinas, Alfonso Aguilera, Mercedes Gameros, Rosa Elena Cuevas Ruiz, Mary H. Hayden, Gerardo Alvarez, Andrew Monaghan, Daniel Williamson, Josh Arnbrister, Eileen Jeffrey Gutiérrez, Yves Carrière, Michael A. Riehle

**Affiliations:** 1 Department of Epidemiology and Biostatistics, College of Public Health, University of Arizona, Tucson, AZ 85721, USA; 2 Department of Entomology, College of Agriculture and Life Sciences, University of Arizona, Tucson, AZ 85721, USA; 3 Centro de Estudios en Salud y Sociedad, El Colegio de Sonora, Hermosillo 83000, Sonora, Mexico; 4 Institute for Health Metrics and Evaluation, University of Washington, Seattle, WA 98195, USA; 5 Veterinary Molecular Biology Laboratory, Instituto Tecnológico de Sonora, Obregon 85059, Sonora, Mexico; 6 Centro de Salud Urbano de Nogales, Nogales 84100, Sonora, Mexico; 7 Lyda Hill Institute for Human Resilience, University of Colorado, Colorado Springs, CO 80918, USA; 8 División de Ciencias Biológicas y de la Salud, Universidad de Sonora, Hermosillo 83000, Sonora, Mexico; 9 Center for Research Data & Digital Scholarship, University of Colorado, Boulder, CO 80309, USA; 10 Divisions of Biostatistics & Epidemiology, School of Public Health, Innovative Genomics Institute, University of California Berkeley, Berkely, CA 94720, USA

**Keywords:** *Aedes aegypti*, longevity, climate, Mexico, age-grading, extrinsic incubation period, parity, mosquito, dengue

## Abstract

Dengue transmission is determined by a complex set of interactions between the environment, *Aedes aegypti* mosquitoes, dengue viruses, and humans. Emergence in new geographic areas can be unpredictable, with some regions having established mosquito populations for decades without locally acquired transmission. Key factors such as mosquito longevity, temperature-driven extrinsic incubation period (EIP), and vector–human contact can strongly influence the potential for disease transmission. To assess how these factors interact at the edge of the geographical range of dengue virus transmission, we conducted mosquito sampling in multiple urban areas located throughout the Arizona–Sonora desert region during the summer rainy seasons from 2013 to 2015. Mosquito population age structure, reflecting mosquito survivorship, was measured using a combination of parity analysis and relative gene expression of an age-related gene, SCP-1. Bloodmeal analysis was conducted on field collected blood-fed mosquitoes. Site-specific temperature was used to estimate the EIP, and this predicted EIP combined with mosquito age were combined to estimate the abundance of “potential” vectors (i.e., mosquitoes old enough to survive the EIP). Comparisons were made across cities by month and year. The dengue endemic cities Hermosillo and Ciudad Obregon, both in the state of Sonora, Mexico, had higher abundance of potential vectors than non-endemic Nogales, Sonora, Mexico. Interestingly, Tucson, Arizona consistently had a higher estimated abundance of potential vectors than dengue endemic regions of Sonora, Mexico. There were no observed city-level differences in species composition of blood meals. Combined, these data offer insights into the critical factors required for dengue transmission at the ecological edge of the mosquito’s range. However, further research is needed to integrate an understanding of how social and additional environmental factors constrain and enhance dengue transmission in emerging regions.

## 1. Introduction

*Aedes aegypti* (L.) (Diptera: Culicidae) is the primary vector of several important arboviruses worldwide, including dengue viruses (DENV), yellow fever virus and Zika virus (ZIKV). Dengue viruses alone threaten almost half of the world’s population [1]. Both the highly invasive mosquito vector and the viruses it can transmit have rapidly expanded their ranges over the past 50 years, resulting in dramatic outbreaks and increased disease burden [2]. The close association between *Ae. aegypti* and humans has facilitated its range expansion, but also typically limits its distribution to urban environments and clustered human dwellings in rural areas [3]. *Aedes aegypti* is considered a tropical/semi-tropical species [4], although some populations may be able to persist in temperate regions using subterranean overwintering habitats [5]. Climate change is likely to further increase the range of this important vector and the arboviruses it transmits [6,7].

While range expansion of vectors raises concern, the presence of the vector mosquito and introduction of an arbovirus are not sufficient to establish disease transmission in new regions [8]. The likelihood of a vector-borne disease outbreak depends on the vectorial capacity of the mosquito population. First developed by MacDonald [9], the concept of vectorial capacity is an estimate of the total number of infectious mosquito bites resulting from a single infected host. The four main components of vectorial capacity are: the length of time between a mosquito consuming an infected bloodmeal and becoming infectious (referred to as the extrinsic incubation period or EIP); the population density of mosquitoes relative to human hosts; female mosquito daily survival and the human biting rate [10]. 

As vector density is a key component of vectorial capacity, vector surveillance is central to arbovirus monitoring and prevention [11]. A systematic review of scientific literature on the correlation between vector indices and dengue cases, however, revealed that quantifiable evidence in favor of these metrics is lacking [12]. Historically, a challenge for predicting arbovirus transmission by *Aedes* spp. has been the lack of efficient adult traps, resulting in the use of larval and pupal surveillance, which are weak predictors of transmission risk and, in some cases, even vector density [13,14]. The development of BG Sentinel traps and various gravid traps now provides better tools for assessing vector density [15]. The peri-domestic habits of *Ae. aegypti*, however, mean even low densities of mosquitoes can cause arboviral outbreaks [16]. Further complicating the use of adult density as an indicator of disease risk, are varying levels of non-human host availability which may support vector populations without amplifying virus [17]. 

Climatic factors such as temperature, humidity, and photoperiod can have complex impacts on vectoral capacity in *Ae. aegypti* that vary by life stage [4]. For instance, Bar-Zeev et al. [18] reported that 32 °C was optimal for larval development time with survival declining at temperatures higher than 40 °C. We determined the optimal temperature for adult survival was lower (27.5 °C), although humidity buffers the impacts of higher temperatures on mortality [19]. Survival past the virus EIP is a critical determinant of transmission potential. The EIP decreases with higher temperatures and transmission has been linked to increased temperature [20]. Chan and Johansson [20] determined that the mean EIP estimate of dengue virus in *Ae. aegypti* decreased from 15 days (95% CI: 10, 20 days) at 25 °C to 6.5 days (95% CI: 4.8, 8.8 days) at 30°C using a log normal model. Similarly, large fluctuations around low temperatures reduced DENV EIP in *Ae. aegypti* by ~36% at a mean of 20 °C [21]. 

Vector management and public health preparedness professionals would benefit from reliable forecasts of mosquito abundance and disease transmission. Current models, while capable of predicting vector density, often fall short of generating reproducible probabilistic forecasts or predicting disease risks [22]. Most early warning systems currently use meteorological alarms, and few have been integrated into routine decision-making [23,24]. More recent developments in predictive risk modeling have begun integrating key biological and physiological factors relevant to mosquito and viral development. Kamiya [25] utilized a stochastic model to examine consequences of temperature variation on the EIP of dengue virus and determined that mathematical models that estimate disease risk are highly temperature sensitive. 

Measuring adult female *Ae. aegypti* survival beyond the EIP is a key factor in predicting and modelling dengue transmission risk. In the MacDonald model, daily probability of mosquito survival has an exponential impact on arboviral transmission, but age-grading wild mosquitoes is difficult. Using molecular techniques developed by Joy et al. [26], an initial study examined the association between mosquito age structure and dengue risk. Older mosquito population were identified in Hermosillo, SN, Mexico, a city with regular dengue transmission, as compared to the mosquito population in Heroica Nogales, SN, a city without significant transmission [27]. The prior study assigned three broad categories of vectors: non-vectors (0–5 days post-emergence), unlikely vectors (6–14 days post-emergence), and potential vectors (15+ days post-emergence). Those estimated >15 days were considered potential vectors by combining the time from eclosion to first blood meal and average EIP. The objective of this current study builds on prior findings to refine the age structure model to estimate mosquitoes age in days and determine whether it exceeds a calculated time-place matched temperature-driven EIP. The study was conducted across a north-south transect from Tucson, Arizona, USA to Ciudad Obregon, Sonora, Mexico which spans the current range of dengue transmission in the region.

## 2. Materials and Methods

### 2.1. Collection Sites

**Figure 1:**
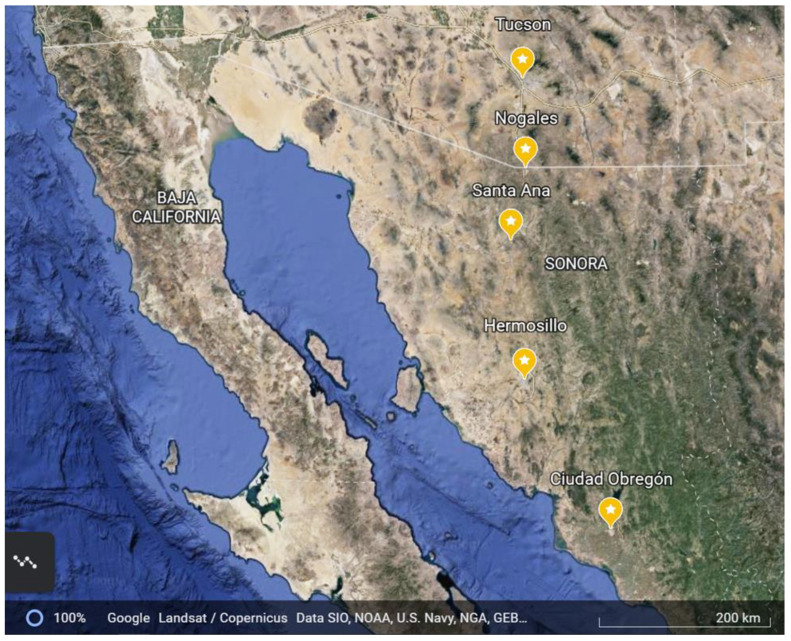
Map of study cities in the Arizona-Sonora Desert region from Arizona (USA) to Sonora (Mexico).

The five cities in the study are in the Sonoran Desert region, which includes southern Arizona (AZ) in the United States and western Sonora (SN), Mexico (Figure 1, Table 1). The region is known for high summer temperatures and limited annual rainfall, most of which occurs during the summer rainy season. The northernmost city is Tucson, Arizona, located approximately 120 km north of the international border between the United States and Mexico. Next is the small border city of Nogales, Arizona. The remaining cities are in the Mexican state of Sonora and include (from north to south) the border city of Nogales immediately south of and adjacent to Nogales, Arizona, Santa Ana, the state capital of Hermosillo and Ciudad Obregon. The latter two cities have endemic dengue transmission.

### 2.2. Climate and Dengue Case Data

Modeled ambient temperatures were obtained for each city at temporal resolutions of 1 hour using North American Land Data Assimilation System (NLDAS) data (https://ldas.gsfc.nasa.gov/nldas (accessed on 31 January 2023)) for the entire study period (July 2013–October 2015). Dengue case information was obtained from the surveillance system of the Health Ministry of the State of Sonora. The state laboratory performed diagnostic confirmation of probable dengue patients depending on timing of specimen collection during clinical disease period of evolution of the infection by using immunosorbent assay (ELISA) for detection of viral antigen NS1 ≥ 1 unit, IgM ≥ 11 units, or high levels of IgG ≥ 22 units as indicator of a recent and active reinfection [28]. Confirmed cases were those with a positive result in one of the two tests. 

### 2.3. Mosquito Sampling

Monthly mosquito sampling took place during the summer rainy season (late July to early October from 2013 through 2015). Mosquito trapping sites were identified within each of the six cities. Traps were placed in outdoor spaces associated with a residence (e.g., on a porch or in a yard). Each site was at least 500 m from other sites. Fewer sites were established in smaller cities due to space limitations. Details of site selection are described in Ernst et al., 2017 [27]. In 2013, 16 sampling sites were established in each of the large cities of Tucson and Hermosillo while 15 sites were established in Nogales, Sonora and five sites were established in Nogales, Arizona. Sampling was expanded into two more cities in Sonora in 2015 to assess the robustness of our findings. We included 15 sites in Ciudad Obregon, SN, a dengue endemic site and ten sites in the smaller town of Santa Ana, located between Hermosillo, SN and Heroica Nogales, SN as representative of a city at high-risk for emergence of dengue. A total of 72 unique mosquito sampling sites were established.

Adult mosquitoes were collected at each site three times during the summer rainy season—once in July or early August, once in late August or early September and once at the end of the wet season in late September or early October. For each trapping event, mosquitoes were collected daily over a 4 day period using a BG Sentinel trap (Bioagents AG, Germany) baited with octenol and a synthetic lure designed to imitate human skin odors [29]. Mosquitoes were removed alive at least once daily and brought to the lab where they were identified to species, separated by sex, and frozen at 80 °C for later parity analysis and age-grading. Adult females found dead in the traps were also collected and included in density measures but were not used for parity and age-grading analyses due to sample degradation. Vector density was determined by the average number of *Ae. aegypti* females collected per site per day during each 4-day trapping event.

### 2.4. Mosquito Parity and Transcription Assessment

Parity was determined for field-collected *Ae. aegypti* as previously described [26,30]. Briefly, ovaries from field-collected female mosquitoes were gently dissected from mosquito abdomens to avoid stretching the tracheal skeins and placed in a drop of deionized water on a clean slide. The water was allowed to evaporate at RT adhering the ovaries to the glass slide. Ovaries were then scanned under an inverted microscope at 100× and 400×. Images were captured using a Spot RT digital camera (Diagnostic Instruments Inc., Sterling Heights, MI, USA) and archived for future reference. Ovaries lacking tracheal skeins, mosquitoes that were gravid and mosquitoes with blood in their midgut were scored as parous. Mosquitoes with multiple tracheal skeins on their ovaries were scored as nulliparous. All live-caught female mosquitoes from each site and trapping event were dissected to determine parity unless over 20 were collected, in which case 20 were randomly selected for analysis.

To determine the approximate age of the field-collected mosquitoes, we assessed the transcript levels of sarcoplasmic calcium-binding protein 1 (SCP-1), a gene showing age-dependent transcription, using quantitative real-time PCR (qPCR) as previously described [26,27]. Following dissections of the ovaries for parity analysis, we stored the head and thorax of the female mosquito in RNAlater. We determined *SCP-1* levels on up to ten of the parous mosquitoes collected per site and trapping event. Previous studies showed that nulliparous mosquitoes nearly always age graded as <5 days old [26]. This was accomplished by isolating total RNA (RNeasy kit, Qiagen, Valencia, CA, USA), converting it into cDNA (High-capacity cDNA kit, Life Technologies, Grand Island, NY, USA) and performing qPCR for *SCP-1* with *RPS17* as a loading control. After calculating ΔCT values, we estimated the mosquitoes age based on an ordinary least squares (OLS) regression model described below. 

### 2.5. Mosquito Blood Meal Analysis

Female *Ae. aegypti* mosquitoes collected with visible bloodmeals in their abdomens were assayed to determine the source of the vertebrate bloodmeal. Due to low numbers of mosquitoes with visible blood meals in smaller cities, analyses were conducted only for Tucson, Heroica Nogales, SN and Hermosillo for all three years. Genomic DNA was isolated from each abdomen using the Qiaamp DNA mini kit (Qiagen, Germantown, MD). The isolated DNA was then subjected to polymerase chain reaction assays using a primer set specific to human cytochrome B (Forward: 5′-GGCTTACTTCTCTTCATTCTCTCCT-3′; Reverse: 5′-TTGCTAGGATGAGGATGGATAGTAA-3’). The PCR conditions were performed using GoTaq (Promega) under the following cycling conditions: 95 °C for 1 min; 95 °C for 30 s, 60 °C for 30 s, 72 °C for 1 min—35 cycles; 72 °C for 10 min. The PCR reactions were size fractionated on a 1.5% agarose gel and visualized with Sybrsafe (Invitrogen, Waltham, MA) under a UVP Geldoc-it system (Analytik Jena US, Upland, CA, USA). All PCR positive samples were scored as human. Negative PCR samples were rescreened using a generic mammalian cytochrome B primer set (Forward: 5′-CCATCCAACATCTCAGCATGATGAAA-3′; Reverse: 5′-GCCCCTCAGAATGATATTTGTCCTCA-3′) following the same cycling parameters as for the human cytochrome B set. The PCR positive samples for the generic mammalian cytochrome B primer set were submitted for Sanger sequencing using the forward primer. The sequences were compared to the NCBI database using blast to identify the source of the bloodmeal. Assays in our lab indicated that we could amplify cytochrome B from the female mosquito within ~24–30 h post-bloodmeal after which the DNA samples were too degraded. Samples that were negative for both human and mammalian cytochrome B were considered to too degraded for successful bloodmeal identification.

### 2.6. Statistical Methods

Differences in *Ae. aegypti* Density and Parity and Bloodmeal hosts: Mean daily female mosquito trap counts were log-transformed to improve assumption of normality. Two-way analyses of variance were used to assess differences in the density of *Ae. aegypti* females among collection cities and months for each year. Due to significant interactions between city and month, the associations between city and mosquito density were further analyzed for each month within a year using a one-way ANOVA with dummy variables and the city Hermosillo, where dengue is transmitted, served as the reference. Logistic regression was used to assess differences in the proportion of parous *Ae. aegypti* females by city and month for each year. The relationship between the proportion of human versus non-human blood meals and the different cities and years was analyzed using a logistic regression model. These statistical analyses were run using JMP (JMP, version 12.1).

**Figure 2:**
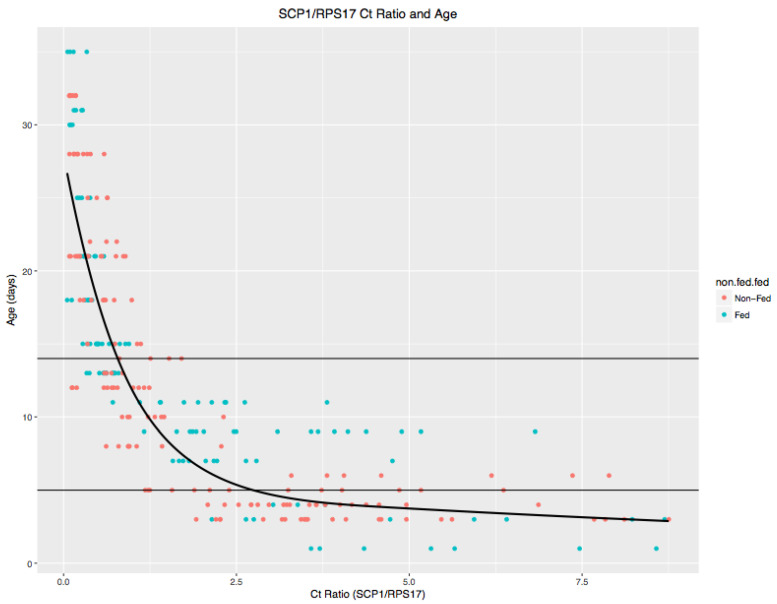
Ct Ratio and Age relationship. The RNA extraction protocol was changed after the initial Joy et al. 2012 paper [26] to eliminate problems caused by extraction from mosquito abdomens. New thresholds were identified (and used in Ernst et al. 2017 [27]): Ct ratio ≥ 1.92: ≤ 5 days old; 0.78 ≤ Ct ratio < 1.92: 6–14 days old; Ct ratio < 0.78: > 14 days old. The new regression model is shown above, with mosquitoes colored by their feeding status. The best-fit linear regression model is shown (bold black line): log(age)~rcs(Ct ratio, 3), where rcs represents a restricted cubic spline with three knots. Fed and non-fed mosquitoes did not differ significantly in their expression profiles (*p* = 0.313; Wald test of regression coefficient for feeding status).

*Age and EIP Estimation:* Statistical analyses were performed in R v3.3.1 software (R Development Core Team, 2016). An ordinary least squares (OLS) regression model was used to relate *SCP-1* expression levels to log-transformed ages (in days) (R package rms; Harrell, 2016). A three-knot restricted cubic spline was used to model the non-linear relationship between *SCP-1* expression and age. This model enabled prediction of mosquito age using transcription data in their continuous form (Figure 2) [31]. 

**Figure 3:**
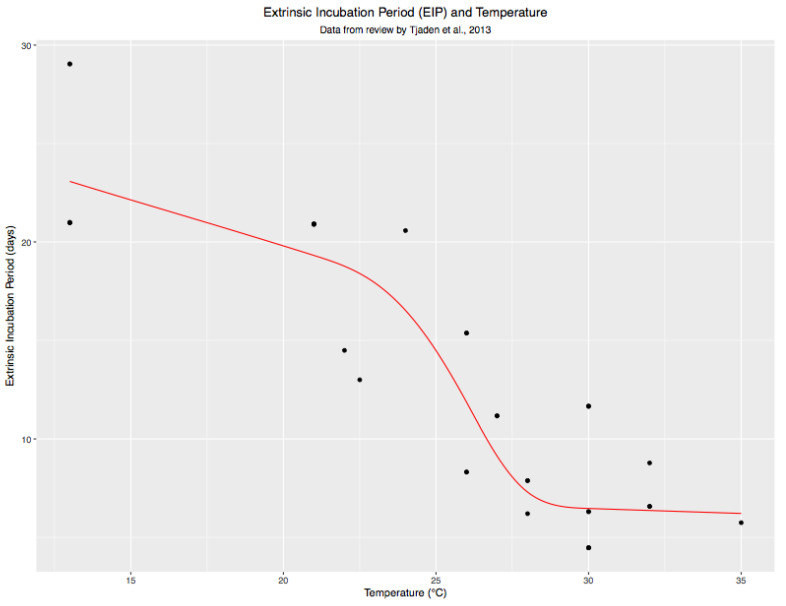
Extrinsic Incubation Period and Temperature model: To account for study-level effects, a mixed effects model was run with random intercepts by study and a 6-knot restricted cubic spline for temperature. The predicted EIP values for the data points used to construct that model were then entered as data in a separate OLS mixed effects model, again with a 6-knot restricted cubic spline for temperature and log transformation of EIP. This model is identical to the previous model in terms of mean estimates, though standard errors are different. The value of this model is in eliminating intra-study variability to visualize average effects estimated by each study.

Chan and Johansson (2012) reviewed published data on the association of temperature with the length of the extrinsic incubation period (EIP) for dengue viruses and used Bayesian mixed effects time-to-event models to quantify this relationship [20]. Employing the data they compiled, we derived a frequentist lognormal parametric time-to-event model with interval censoring [31], relating temperature to the length of the EIP (Figure 3).

Monte Carlo simulation was used to estimate the probability that the age of each individual age-graded *Ae. aegypti* female exceeded the dengue EIP at the temperatures experienced by that mosquito (Figure 2). A total of 100 simulation replicates were performed for each mosquito. This approach accommodated uncertainty in both age and EIP estimates to predict mosquito vector potential. For each replicate, an age estimate was randomly drawn from the prediction interval of the transcription–age model. Davis (1984) examined the onset of feeding by *Ae. aegypti* females in laboratory conditions and presented a linear regression model relating age since eclosion to the probability of feeding onset [32]. We subtracted the median age of feeding onset estimated from their regression model (65.5 h, or 2.73 days) from each simulated age prediction to estimate the length of the realized post-feeding incubation time, *x*. Working backward from the date on which the mosquito was collected (*t*_0_), average daily temperatures were calculated for each post-feeding day (*t_-_*_1_…*t*_-x_) as the mean of hourly temperature estimates from modeled NLDAS data for that date and site. The length of the EIP was randomly drawn from the prediction interval of the lognormal time-to-event model using these temperature values. The inverse of the EIP estimate represents that day’s contribution to the hypothetical total development of dengue viruses in the mosquito. Summing these values over *t*_-1_*–t*_-x_ (with prorated contributions from partial days) yielded the number of EIPs completed by the mosquito. The proportion of simulations in which a mosquito’s estimated age exceeds or equals its estimated EIP (plus 2.73 days) represents the probability that the mosquito was old enough to be a potential dengue vector. An analogous process, going forward in time from the collection date, was used to estimate hypothetical EIP lengths for mosquitoes that had not exceeded their EIP.

*Dengue Incidence:* Dengue incidence was calculated for each municipality by dividing the number of suspected or confirmed cases by the total population of the municipality from the 2010 Mexican census. 

Differences between city-level *Ae. aegypti* Age and EIP: Simulated age and estimated EIP for time of mosquito collections was compared among cities by each year of collection. Median and interquartile range were calculated for mosquito age and estimated extrinsic incubation period by city and by year. Differences among cities were assessed within each year using Wilcoxon rank sum test with continuity correction. 

Differences in Vector Potential: Simulated mosquito ages and EIP lengths were used to estimate differences in risk of exposure to potentially infectious *Ae. aegypti* by collection city, month, and year, and aggregated by year. For each trapping site and month, the vector potentials (probabilities of exceeding EIP) of all age-graded mosquitoes were summed to derive an average total number of possible vectors. This value was multiplied by the proportion of female *Ae. aegypti* that were parous and by the total number of female *Ae. aegypti*, to estimate the number of potential vectors at that site and month. Negative binomial regression models (R MASS package; [33]) were used to calculate incidence rate ratios (IRRs) for counts of potential vector, with log-transformed counts of trap days per event as an offset in the model, to account for differences in trapping effort. Differences among cities within a given month and year were evaluated using stratification by month and year, and with city as a predictor. Cumulative seasonal comparisons were made by aggregating data by year. Temporal differences in vector density within individual cities were evaluated using similar models, with stratification by city and with month, year, and their interaction as predictors.

## 3. Results

### 3.1. Mosquito Density and Parity 

A total of 8,307 *Ae. aegypti* females were collected from the 72 trapping sites in six cities over the three years of the study. *Aedes aegypti* female density varied significantly between cities in all three years and between seasons in 2013 and 2014 (Figure 3, Supplementary Table S1). The city of Tucson exhibited significantly higher mosquito counts than the reference city, Hermosillo (2013 early season—t ratio = 3.64 *p* = 0.0007, mid-season—t ratio = 3.84, *p* = 0.0004, late season—not significant; 2014 early—n.s., mid—t ratio = 7.41, *p* < 0.0001, late—t ratio = 2.60, *p* = 0.012; 2015 early—t ratio = 2.30, *p* = 0.024, mid—t ratio = 3.86, *p* = 0.0002, late—t ratio = 7.63, *p* < 0.0001). 

**Figure 4:**
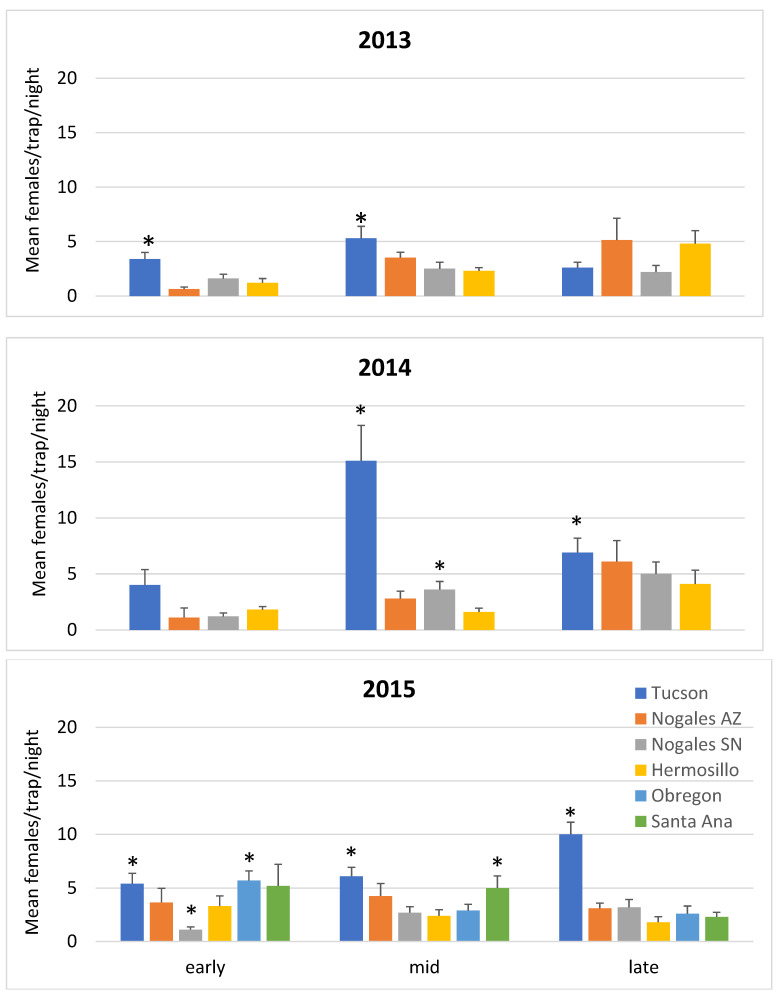
Average female mosquito trap catches by city and sampling period (early = 15 July to 14 August; mid = 15 August to 14 September; late = 15 September to 14 October) for years 2013 to 2015. An asterisk * denotes a city with mosquito counts that are significantly different (*p* < 0.05) from the reference city, Hermosillo (in yellow).

**Figure 5:**
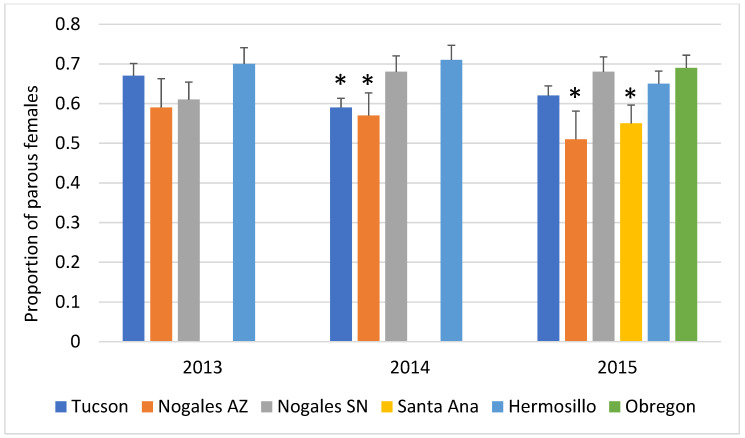
Proportion of parous mosquitoes by city and year. The asterisk * denotes cities with significantly different parity levels (*p* < 0.05) than the reference city, Hermosillo (in light blue).

Approximately 33% of the collected female mosquitoes (2745 individuals) were dissected to determine parity status. Parity rates were consistently over 50% in all the larger cities—Tucson, Nogales SN, Hermosillo, SN and Obregon, SN (Figure 4). There were no significant differences in the proportions of parous mosquitoes across cities or seasons in 2013. In 2014, a significant association between parity levels and city was observed (*p* = 0.0018) due to lower parity levels in Tucson, AZ and Nogales, AZ, but there was not a significant effect of season or significant interaction between city and season. Again in 2015, parity levels varied significantly across cities (*p* >0.0001) but not seasons. A significant interaction between the two factors was also observed (*p* = 0.023) (Figure 5).

### 3.2. Dengue Incidence

No dengue was reported in the Arizona sites during the study period. In Mexico, dengue incidence was highest in October of all three years. In 2014, locally acquired cases of dengue were identified in Heroica Nogales, SN. Incidence in Hermosillo, SN was highest in 2014 with 79 per 100,000 confirmed cases and 196 per 100,000 suspected cases. Obregon, SN and Santa Ana, SN also had documented transmission in October 2015; 15 per 100,000 and 6 per 100,000 confirmed case incidences, respectively (Table 2). 

### 3.3. Mosquito Blood Meal Analysis

**Figure 6:**
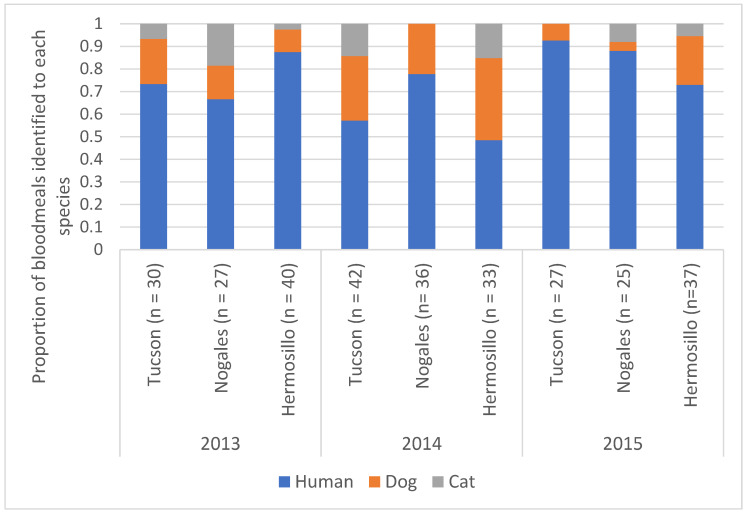
Bloodmeal analysis conducted on engorged female *Ae. aegypti* mosquitoes from trapping events in Tucson, AZ, Hermosillo, SN, and Heroica Nogales, SN from 2013–2015. The proportion of mosquitoes found with blood of each mammalian species. No significant differences by city. 2014 had significantly less human bloodmeals than the other years.

A total of 388 female *Ae. aegypti* with detectable blood in their abdomen were assessed for the source of the bloodmeal (Hermosillo *n* = 138, Nogales *n* = 120 and Tucson *n* = 130). Of these we were able to successfully PCR amplify the cytochrome B amplicon from over 75% of the samples (Hermosillo *n* = 109, Nogales *n* = 89 and Tucson *n* = 100). A majority of the bloodmeals were identified as human in origin (Figure 6), with domestic dogs being the next most abundant and domestic cats representing the rest of the samples. There was no significant difference in the proportion of human blood meals between the three cities tested (Tucson, Nogales SN and Hermosillo) (*p* = 0.12) but the odds of human blood meals were significantly lower in 2014 (*p* = 0.0136) (Figure 6). 

### 3.4. Variations in Average EIP by City and Time Period

The temperature driven EIP was estimated across each sampling month and year (Table 3 and Table S1). Fluctuations were identified between and within years and sites. In all instances except for July to August 2015 in Tucson, AZ 9.6 days (July) and 9.1 days (August), the estimated EIP lengthened from across the months, with July having the shortest estimated EIP and September having the longest EIP. The estimated EIP differed significantly across the study sites for each year. The estimated EIP was similar in multiple years within Tucson, AZ (2014: 9.9, 2015: 9.7), Hermosillo (2013: 9.4, 2014: 10.3, 2015: 9.7), Santa Ana (2015: 10.1) and Obregon, SN (2015: 9.6), with all estimates approximately 10 days. Nogales, AZ and Heroica Nogales, SN estimates were similar to each other but varied among the years. In 2013, the estimated EIP was 15.6 for Heroica Nogales, SN and 16.8 for Nogales, AZ, it was longer in 2014 (20.2, Heroica Nogales, SN and 18.6 Nogales, AZ) and shorter in 2015 (12.9 Heroica Nogales, SN and 12.7 Nogales, AZ) (Table 3).

### 3.5. Differences between City-Level Ae. Aegypti Age

Median estimated mosquito age was significantly older in Hermosillo, SN in 2013 (6.9 days (4.3, 12.8) as compared to Heroica Nogales, SN (5.6 (4.0, 8.5) *p* < 0.001) and similar to that of mosquitoes in Nogales, AZ (6.5 (4.2, 12.6), *p* = 0.11) Table 3). In 2014, Tucson, AZ had the oldest mosquitoes (10.1 (5.6, 14.4)) which were significantly older than those of Heroica Nogales, SN (*p* < 0.01). Heroica Nogales, SN and Hermosillo, SN had equivalent age, (7.8 (5.3, 11.8) and (7.3 (4.7, 12.4) *p* = 0.47), respectively. Mosquitoes in Nogales, AZ were significantly younger than those in Heroica Nogales, SN (5.0 (3.9, 8.6) *p* < 0.001). During 2015, overall mosquito ages were younger than in 2014. Heroica Nogales, SN had the oldest mosquitoes (6.4 (4.4, 10.5), which was significantly older than the estimated age of mosquitoes in Nogales, AZ (4.6 (3.8, 6.8) *p* < 0.001, Santa Ana, SN (5.0 (4.2, 7.7) *p* = 0.01, and Obregon, SN (5.3 (4.2, 8.4) *p* = 0.01 (Table 3). 

### 3.6. Proportion of Mosquitoes That Survived Past the EIP

The median probability that a mosquito exceeds the EIP was less than 0.02 in all three years for Heroica Nogales, SN (2013: 0.01, 2014: 0.01, and 2015: 0.02), very similar to Nogales, AZ (2013: 0.03, 2014: 0.00, 2015: 0.00). Tucson, AZ (2014: 0.51, 2015: 0.13) and Hermosillo, SN (2013: 0.24, 2014: 0.21, 2015: 0.13) had the highest probability of mosquitoes exceeding the EIP. Both Santa Ana, SN (2015: 0.06) and Obregon, SN (2015: 0.08) had lower probabilities of mosquitoes exceeding the EIP than Hermosillo, SN or Tucson, AZ (Table 3).

### 3.7. Estimated Potential Vectors Per Trap Night

The estimated numbers of potential vectors per trap night were generally highest for Tucson, AZ followed by Hermosillo, SN and Obregon, SN (Table 3). Each year, the peak abundance month for Tucson, AZ was August (2013: 1.21, 2014: 3.74, 2015: 1.46) (Table S1). Heroica Nogales, SN, also peaked primarily in August, however the number of potential vectors per day was much lower (2013: 0.22, 2014: 0.23, 2015: 0.37). Hermosillo, SN had peak abundance of potential vectors later in September in 2013 (1.30) and 2014 (0.59), but this trend reversed in 2015 where July had the highest (0.68) followed by August (0.48) and September (0.13). Obregon, SN also peaked in July that year and was slightly above Hermosillo (July: 0.97, August 0.43, and September 0.43) (Table S1).

### 3.8. Differences in Vector Potential (IRR)

The incidence rate ratio compared cities within the transect to Heroica Nogales, SN as the hypothesized lowest risk city (Table 3). In 2013, there was no significant difference in July, but in August, Tucson, AZ and Hermosillo, SN were higher (Tucson IRR = 5.2 (2.5, 11.2) and Hermosillo, SN 2.2 (1.0, 4.9)) (Table S1). This persisted in September with an even higher IRR for Hermosillo 11.0 (3.3, 40.5), which corresponded with the peak number of cases that year in Hermosillo (*n* = 7) (Table S1). When examining the aggregate data for all three months, the overall IRR was 4.0 (2.3, 7.2) for Hermosillo, SN compared to Heroica Nogales, SN. In 2014, only Tucson, AZ had an IRR over 1.0 consistently across all months (July: 3.7 (1.5, 9.5), August 17.2 (8.3, 37.4), and September 6.0 (2.4, 15.6)) (Table S1). Interestingly, Hermosillo, which had more reported cases in 2014 (*n* = 434 cases), did not achieve a significant incidence rate ratio in July or August, though it approached significance in September 2.2 (0.9, 5.1) and the overall annual IRR for 2014 was 1.9 (1.2, 3.4). In 2015, the season started with higher IRR, Tucson, AZ 6.8 (2.6, 21.0), Santa Ana, SN 3.1 (1.0, 10.0), Hermosillo, SN 6.7 (2.6, 20.6), and Obregon, SN 9.9 (3.9, 30.4). The IRR declined over the season but Obregon, SN and Tucson, AZ had significant IRR in September. Tucson, AZ, Obregon, SN, and Hermosillo, SN, all were significantly higher when aggregating across the sampling months in 2015. (Table 3)

## 4. Discussion

Understanding the factors driving differential dengue transmission is important to predicting the risk of emergence of viral transmission in new geographic areas. Within the Arizona–Sonora border region, dengue has generally been restricted to southern Sonora, MX despite established *Ae. aegypti* populations throughout all urban areas within the region. In the current study, we examined regional differences in mosquito abundance and parity. We further introduced two novel indicators of transmission potential; weather-driven estimates of the extrinsic incubation period and the number of potential vectors per trap night based on estimated age exceeding the calculated EIP from the collection period. We found significant intra-annual, inter-annual and inter-city variability in mosquito indices throughout urban areas in the Arizona–Sonora border region. Correlation with reported dengue cases varied by indicator as discussed below. 

Vector abundance has long been discussed as a potential indicator of dengue transmission risk [34], however current evidence is mixed. Our results demonstrate no significant differences in female mosquito counts or parity levels between cities with and without dengue transmission. The lack of association between vector density, blood feeding success and dengue risk we found is consistent with results observed in Brazil [35]. Several studies, however, have identified positive associations between vector abundance and dengue transmission at smaller scales [36,37]. Our trapping locations were limited and might not represent the overall abundance levels throughout the cities. Follow-up analyses to investigate the number of dengue cases reported around specific trap sites or increasing the number of trapping locations within a city could improve the correlation between *Ae. aegypti* abundance and dengue transmission at a city-level. While parity overall was not significantly different among the cities we studied, during the dengue outbreak year of 2014, the proportion of parous female mosquitoes was higher in the two cities where dengue transmission occurred (Hermosillo, SN and at least one locally acquired case in Heroica Nogales, SN). 

We estimated significant differences in the patterns of temperature driven EIP across the study area. The adjacent cities of Heroica Nogales, SN and Nogales, AZ are at higher elevation and have lower average temperatures than the other cities investigated. During the hottest month of July, the estimated EIP differed by approximately four days between all the study cities. However, during early fall, when transmission typically peaks in the region, the EIP was nearly two weeks longer in Ambos Nogales as compared to the other cities. This pattern was relatively consistent across all three years. The longer EIP during the peak transmission months could contribute to the noted differences in dengue transmission between Heroica Nogales, SN, which has equivalent or more abundant *Ae. aegypti* populations and a high social-vulnerability index [38] compared to endemic Hermosillo, SN. Warming temperatures could reduce the EIP and increase the potential risk of transmission of dengue, or other *Aedes*-borne arboviruses with a shorter EIP, such as chikungunya virus [39].

Cities that had a dengue incidence rate greater than 10 per 100,000 (Hermosillo, SN and Ciudad Obregon, SN) also had a higher estimated number of potential vectors per trap night than Heroica Nogales, SN. This correlation was not, however, consistent across the transect. Tucson, AZ, for example, had the highest number of estimated vectors per trap night yet has no locally confirmed cases of dengue. Previous efforts to understand binational differences in dengue risk in Texas suggest that factors such as air conditioning, and more impervious housing structure may limit vector-human contact [40,41]. Blood meal analyses, however, did not demonstrate any significant difference in host patterns between Hermosillo, SN, Tucson, AZ, and Nogales. The majority of bloodmeals were of human origin, with dogs and cats as additional hosts. The sample size of mosquitoes with blood meals still undigested was relatively low and accounted for less than 10% of mosquitoes collected within those sites. Binational differences may also be partially attributed to non-detection bias. Providers in southern Arizona have poor knowledge of dengue and are unlikely to test even individuals with symptoms consistent with dengue infection [42]. Longer term cohort studies are required to assess the multi-factorial determinants of these binational disparities in dengue transmission.

The scale of the current study may limit our ability to find strong and consistent correlations between dengue incidence and our estimate of potential vectors per trap night. The estimated flight range of *Ae. aegypti* mosquitoes is approximately 100 m [43]. Our sampling strategy placed at least 500 m between trapping sites. While this created independent trapping observations, it may not have accurately captured variation throughout the cities. Furthermore, placement was based on geographical distance and not human population density. Dengue cases are more likely to arise from foci of higher human population density [44]. Further investigation into correlations of abundance, age structure, and dengue incidence is warranted at finer spatial scales.

The age structure of local *Ae. aegypti* populations is among the most influential components of vectorial capacity. Mark-release-recapture studies are limited by reliance on laboratory-reared *Ae. aegypti*, challenges in simultaneous assessment under different climatic conditions, and the ethics of releasing females in areas with potential viral transmission [45,46]. Age-grading field caught mosquitoes can be an important tool to understand transmission potential, particularly in areas where *Ae. aegypti* are established, but transmission has not yet been detected (16). Limited studies have been conducted to apply qPCR-based age grading techniques to field mosquitoes, however they demonstrate significant promise in developing a more robust understanding of the complex factors underlying potential disease emergence. Hugo et al. identified an association between age structure and DENV season onset and cessation [47]. This is supported by our results which indicate an increase in mosquito age in September and October, prior to the onset of dengue transmission. Our current analyses also demonstrate the limits of our previous assignment of mosquitoes aged greater than 15 days as potential vectors. In Hermosillo, where the EIP plus average time to first blood meal was under 10 days, this technique significantly underestimated the abundance of mosquitoes that could have survived the EIP. However, in cooler cities where the EIP could be as long as 30 days, it grossly overestimated the abundance.

There are several limitations that should be noted about the current study. Collections were conducted in a limited number of study sites for each of the urban areas. In particular, the Nogales, AZ sites in July 2013 only yielded a total of 10 mosquitoes. Therefore, yearly summaries are likely the most robust in identifying city-level variation as this includes a minimum of 182 mosquitoes. These collections may not fully represent the *Ae. aegypti* population age structure for the city. Studies with more sites are needed to determine if these results are consistent beyond the three years and sites included in this study. Further, we collected mosquitoes for three one-week periods, instead of throughout the entire mosquito season. The timing between precipitation events and mosquito collections may have differed among the cities. If large hatchings occurred following these rainfall events, this could lead to a younger age structure for those conducted within a week following a rainfall event compared to population sampling that occurred at longer time points from the rainfall event. Our dengue case data was for the entirety of the municipalities. We were further resource constrained by the number of SCP-1 analyses that could be conducted. We randomly selected up to 10 females for testing from each collection. This may have under- or over-estimated age if there is a significant correlation between density and longevity. The SCP-1 expression as a single determinant of age within our samples, may be limited in accuracy. Our previous work indicated that the SCP-1 gene expression may not perform well for the oldest mosquitoes; those >35 days old. This may limit results for Heroica Nogales, SN, where the estimated EIP was 30 days in 2014. Identifying additional genes to include in a multivariate model could strengthen our age-grading method. Refining analyses to households within a 100 m radius of trapping locations may have yielded stronger correlations between transmission and *Ae. aegypti* indices. Finally, we are seeking permission to obtain geo-coded case information to explore these relationships further. 

## 5. Conclusions

Our results support the potential for using age-grading and estimations of the extrinsic incubation period to better understand risk of transmission in areas at risk for dengue emergence. More refined age-grading assessments combined with an estimation of the temperature-driven extrinsic incubation period are needed. However, our results also illustrate the complexity of determinants of disease transmission. Tucson, AZ, was identified as the highest risk urban area within the region based on vector indices, yet there have been no confirmed locally acquired cases of dengue identified there. Our study was limited by the number of sites and mosquitoes we were able to test. Recent identification of the first locally-acquired dengue case in Phoenix, AZ, highlights the need to be vigilant. Active surveillance and healthcare provider education should be emphasized to detect potential locally acquired cases. 

## Figures and Tables

**Table 1:** Climate averages among study site cities.

City	Population ^1^	Elevation (m Above Sea Level)	Average ^2^ Rainfall (mm)	Average High/Low July Temperature (°C)	Average High/Low August Temperature (°C)	Average High/Low September Temperature (°C)
Tucson, AZ	520,561	807	294	38.3/24.2	33.3/23.9	35.0/21.7
Nogales, AZ	19,766	1167	460	34.4/19.4	36.1/16.7	31.1/11.1
Heroica Heroica Nogales, SN	212,533	1214	455	34.1/17.0	33.5/19.3	32.1/18.5
Santa Ana, SN	11,864	687	327	39.0/19.0	38.0/21.4	36.8/21.0
Hermosillo, SN	715,061	210	387	39.8/23.8	38.3/25.6	38.3/25.6
Ciudad Obregon, SN	298,625	40	223	38.3/23.3	38.5/25.5	38.4/25.2

^1^ Population data for Mexican cities based on 2010 census (INEGI 2015). Tucson data based on 2010 census (US Census 2015); ^2^ Rainfall and temperature averages for Mexico are from the Servicio Meteorologica Nacional (Mexico) database (SMN 2023). Tucson climate data are from the (U.S.) National Weather Service (NWS 2023); temperature based on climate normals (1991–2020).

**Table 2:** Dengue incidence by municipality during and one month following the end of the sampling period for all sites. Confirmed cases (left) and suspected cases (right). No data presented for Tucson, AZ or Nogales, AZ where no locally acquired dengue cases were reported during the study period 2013–2015.

Cases per 100,000—Confirmed						Cases per 100,000—Suspected				
	Heroica Nogales, SN	Santa Ana, SN	Hermosillo, SN	Obregon, SN			Heroica Nogales, SN	Santa Ana, SN	Hermosillo, SN	Obregon, SN
2013 July	0.00		0.24			13 July	0.00		1.22	
2013 August	0.00		0.12			13 August	0.00		1.22	
2013 September	0.00		0.98			13 September	0.00		2.93	
2013 October	0.46		5.12			13 October	0.91		14.63	
2014 July	0.46		0.49			14 July	0.91		1.34	
2014 August	0.00		1.95			14 August	0.00		2.80	
2014 September	0.91		4.63			14 September	1.37		18.54	
2014 October	4.56		79.02			14 October	4.56		195.61	
2015 July	0.00	0	0.98	1.85		15 July	0.00	0	1.59	2.46
2015 August	0.00	0	1.83	1.85		15 August	0.46	0	6.22	9.23
2015 September	0.46	0	12.32	4.62		15 September	0.91	0	43.05	9.23
2015 October	1.37	6.15	57.32	15.39		15 October	2.28	6.15	120.73	22.16

**Table 3:** Comparison of estimated age of parous mosquitoes, estimated extrinsic Incubation period + average days post eclosion, the probability that a given mosquito will exceed the EIP, and the number of potential vectors per trap per day, and the incident rate ratio of potential vectors per day. Heroica Nogales, SN is used as standard for all statistical comparisons. Ref. = Reference city. Details for each month in Supplementary Table S1.

Year	City	Total N	Age of Parous Mosquitoes, in Days, Median (Q1, Q3)	*p*-Value (Age) ^a^	EIP + 2.63 Days, Median (Q1, Q3)	*p*-Value (EIP) ^b^	Prob. Exceeds EIP, Median (Q1, Q3)	No. Potential Vectors/Trap/Day	IRR (95% CI) ^c^
2013	Heroica Nogales, SN	370	5.6 (4.0, 8.5)	Ref.	15.6 (13.1, 22.0)	Ref.	0.01 (0.00, 0.12)	0.2	Ref.
	Tucson *	-	-	-	-	-	-	-	-
	Nogales, AZ	182	6.5 (4.2, 12.6)	0.11	16.8 (12.9, 21.1)	0.86	0.03 (0.00, 0.27)	0.4	2.1 (0.9, 4.9)
	Hermosillo	599	6.9 (4.3, 12.8)	<0.001	9.4 (9.1, 10.0)	<0.001	0.24 (0.04, 0.75)	0.8	4.0 (2.3, 7.2)
2014	Heroica Nogales, SN	586	7.8 (5.3, 11.8)	Ref.	20.2 (15.4, 28.6)	Ref.	0.01 (0.00, 0.17)	0.25	Ref.
	Tucson	1665	10.1 (5.6, 14.4)	<0.01	9.9 (9.4, 12.0)	<0.001	0.51 (0.11, 0.77)	2.12	8.4 (5.0, 14.3)
	Nogales, AZ	200	5.0 (3.9, 8.6)	<0.001	18.6 (15.4, 31.1)	0.55	0.00 (0.00, 0.02)	0.07	0.3 (0.1, 0.8)
	Hermosillo	931	7.3 (4.7, 12.4)	0.47	10.3 (10.0, 10.8)	<0.001	0.21 (0.03, 0.68)	0.43	1.8 (1.1, 2.9)
2015	Heroica Nogales, SN	417	6.4 (4.4, 10.5)	Ref.	12.9 (12.6, 19.6)	Ref.	0.02 (0.00, 0.19)	0.2	Ref.
	Tucson	1344	5.9 (4.1, 9.7)	0.15	9.7 (9.3, 10.6)	<0.001	0.13 (0.03, 0.49)	1.1	5.1 (3.3, 8.0)
	Nogales, AZ	220	4.6 (3.8, 6.8)	<0.001	12.7 (12.1, 20.1)	0.1	0.00 (0.00, 0.05)	0.1	0.4 (0.2, 1.1)
	Santa Ana	500	5.0 (4.2, 7.7)	0.01	10.1 (9.8, 11.1)	<0.001	0.06 (0.02, 0.24)	0.4	1.5 (0.9, 2.6)
	Hermosillo	469	5.8 (4.2, 9.7)	0.34	9.7 (9.1, 10.7)	<0.001	0.13 (0.02, 0.50)	0.4	1.9 (1.2, 3.1)
	Obregon	674	5.3 (4.2, 8.4)	0.01	9.6 (9.3, 9.9)	<0.001	0.08 (0.03, 0.36)	0.6	2.7 (1.7, 4.3)

^a^ Logistic regression for binomial proportions ^b^ Wilcoxon rank sum test with continuity correction ^c^ Negative binomial regression model stratified by collection cycle and year, relating count of potential vectors per household collection site with log (trap days) as offset and with city as a predictor. * Ref. indicates the city used for comparisons. July Tucson 2013 missing due to differing RNA extraction methods used for mosquito samples during that collection period.

## Data Availability

Data will be made publicly available through the University of Arizona Research Data Repository (ReDATA).

## Supplementary Materials

The following supporting information can be downloaded at: https://www.mdpi.com/article/10.3390/v15040851/s1, Table S1: Monthly indices for Ae. aegypti.
