# Supplementary material for: Differences in Longevity and Temperature-Driven Extrinsic Incubation Period Correlate with Varying Dengue Risk in the Arizona–Sonora Desert Region
Source: Viruses. 2023 Mar 26;15(4):851. doi: 10.3390/v15040851 (PMC10146351; doi:10.3390/v15040851)
Supplement: Supplementary file 1 [file viruses-15-00851-s001.zip › viruses-2222976-supplementary.pdf]

Table S1. Comparison of estimated age of parous mosquitoes, estimated extrinsic Incubation period + average days post eclosion, the probability that a given mosquito will exceed the EIP, and the number of potential vectors per trap per day, and the incident rate ratio of potential vectors per day. Nogales, SN is used as standard for all statistical comparisons.

**Year**	**Month**	**City**	**Total N**	**Age of Parous Mosquitoes, in days, median (Q1, Q3)**	***P*-value (age)** [a]	**EIP + 2.63 days, median (Q1, Q3)**	***P*-value (EIP)** [b]	**Prob. Exceeds EIP, median (Q1, Q3)**	**No. Potential Vectors/ trap/day**	**IRR (95% CI)** [c]
**2013**	Jul	Nogales, SN	96	4.1 (3.4, 6.5)	Ref.	12.9 (12.7, 13.1)	Ref.	0.00 (0.00, 0.06)	0.16	Ref.
		Tucson*	-	-	-	-	-	-	-	-
		Nogales, AZ	10	20.2 (19.8, 21.8)	**< 0.01**	13.6 (13.6, 13.6)	**<0.001**	0.85 (0.82, 0.86)	0.21	1.3 (0.3, 6.2)
		Hermosillo	73	9.0 (4.8, 15.3)	**< 0.001**	8.8 (8.5, 9.1)	**<0.001**	0.50 (0.08, 0.88)	0.42	2.1 (0.8, 5.8)
**2013**	Aug.	Nogales, SN	143	5.2 (4.0, 8.8)	Ref.	15.5 (14.0, 16.3)	Ref.	0.02 (0.00, 0.21)	0.22	Ref.
		Tucson	338	8.4 (5.4, 14.6)	**< 0.001**	10.7 (10.3, 10.9)	**<0.001**	0.33 (0.07, 0.80)	1.21	**5.2 (2.5, 11.2)**
		Nogales, AZ	70	5.3 (3.8, 8.2)	0.83	12.9 (12.4, 15.9)	**<0.001**	0.03 (0.00, 0.18)	0.32	1.3 (0.4, 4.1)
		Hermosillo	145	7.3 (4.9, 11.7)	**0.01**	9.3 (9.1, 9.4)	**<0.001**	0.30 (0.08, 0.72)	0.52	**2.2 (1.0, 4.9)**
**2013**	Sept/ Oct	Nogales, SN	131	6.7 (5.1, 8.9)	Ref.	30.4 (22.6, 41.8)	Ref.	0.01 (0.00, 0.09)	0.08	Ref.
		Tucson	167	6.3 (4.6, 13.8)	0.6	14.3 (11.3, 18.4)	**<0.001**	0.09 (0.02, 0.67)	0.55	**4.7 (1.4, 17.7)**
		Nogales, AZ	102	7.2 (4.4, 12.8)	0.59	21.8 (18.4, 31.0)	**0.01**	0.02 (0.00, 0.26)	0.56	**5.3 (1.2, 27.7)**
		Hermosillo	381	6.6 (4.1, 12.7)	0.84	10.0 (9.8, 10.2)	**<0.001**	0.15 (0.01, 0.73)	1.30	**11.0 (3.3,40.5)**
**2013**		Nogales, SN	370	5.6 (4.0, 8.5)	Ref.	15.6 (13.1, 22.0)	Ref.	0.01 (0.00, 0.12)	0.2	Ref.
		Tucson	-	-	-	-	-	-	-	**-**
		Nogales, AZ	182	6.5 (4.2, 12.6)	0.11	16.8 (12.9, 21.1)	0.86	0.03 (0.00, 0.27)	0.4	2.1 (0.9, 4.9)
		Hermosillo	599	6.9 (4.3, 12.8)	**<0.001**	9.4 (9.1, 10.0)	**<0.001**	0.24 (0.04, 0.75)	0.8	**4.0 (2.3, 7.2)**
**2014**	Jul	Nogales, SN	71	8.0 (5.0, 11.9)	Ref.	13.5 (12.1, 14.6)	Ref.	0.20 (0.01, 0.55)	0.27	Ref.
		Tucson	259	9.5 (5.8, 13.6)	0.46	9.4 (9.2, 9.7)	**<0.001**	0.54 (0.12, 0.79)	1	**3.7 (1.5, 9.5)**
		Nogales, AZ	22	3.9 (3.3, 4.4)	**<0.001**	15.2 (14.5, 16.2)	**<0.01**	0.01 (0.00, 0.02)	0.01	n/a
		Hermosillo	223	7.7 (4.8, 13.8)	0.68	9.9 (9.7, 10.0)	**<0.001**	0.32 (0.05, 0.78)	0.42	1.5 (0.6, 3.3)
**2014**	Aug.	Nogales, SN	213	6.7 (4.5, 11.9)	Ref.	16.0 (15.2, 18.1)	Ref.	0.00 (0.00, 0.08)	0.23	Ref.
		Tucson	964	10.3 (5.8, 14.3)	**0.02**	9.6 (9.4, 10.1)	**<0.001**	0.57 (0.13, 0.82)	3.74	**17.2 (8.3, 37.4)**
		Nogales, AZ	56	5.7 (4.4, 8.7)	0.26	15.5 (15.0, 16.8)	0.07	0.00 (0.00, 0.02)	0.06	0.2 (0.0, 1.4)
		Hermosillo	189	8.6 (5.5, 12.3)	0.12	10.3 (10.2, 10.4)	**<0.001**	0.32 (0.07, 0.66)	0.28	1.5 (0.7, 3.3)
**2014**	Sept/ Oct	Nogales, SN	302	8.1 (5.8, 11.0)	Ref.	28.8 (25.6, 31.1)	Ref.	0.01 (0.00, 0.07)	0.25	Ref.
		Tucson	442	10.3 (4.8, 14.7)	0.29	13.0 (12.3, 15.4)	**<0.001**	0.39 (0.02, 0.69)	1.62	**6.0 (2.4, 15.6)**
		Nogales, AZ	122	5.4 (3.8, 11.6)	**<0.01**	31.3 (25.1, 35.6)	**0.04**	0.00 (0.00, 0.06)	0.13	0.6 (0.1, 2.9)
		Hermosillo	519	6.4 (4.5, 10.7)	**<0.01**	10.8 (10.5, 11.0)	**<0.001**	0.13 (0.02, 0.52)	0.59	2.2 (0.9, 5.1)
**2014**		Nogales, SN	586	7.8 (5.3, 11.8)	**Ref.**	20.2 (15.4, 28.6)	**Ref.**	0.01 (0.00, 0.17)	0.25	Ref.
		Tucson	1665	10.1 (5.6, 14.4)	**<0.01**	9.9 (9.4, 12.0)	**<0.001**	0.51 (0.11, 0.77)	2.12	8.4 (5.0, 14.3)
		Nogales, AZ	200	5.0 (3.9, 8.6)	**<0.001**	18.6 (15.4, 31.1)	0.55	0.00 (0.00, 0.02)	0.07	0.3 (0.1, 0.8)
		Hermosillo	931	7.3 (4.7, 12.4)	0.47	10.3 (10.0, 10.8)	**<0.001**	0.21 (0.03, 0.68)	0.43	1.8 (1.1, 2.9)

**2015**	Jul	Nogales, SN	66	5.4 (4.0, 8.1)	Ref.	11.8 (11.6, 12.3)	Ref.	0.02 (0.00, 0.12)	0.08	Ref.
		Tucson	314	4.7 (3.8, 7.3)	0.24	9.6 (9.5, 9.7)	**<0.001**	0.05 (0.01, 0.26)	0.61	**6.8 (2.6, 21.0)**
		Nogales, AZ	73	4.7 (3.8, 6.7)	0.32	11.7 (11.5, 12.1)	0.28	0.01 (0.00, 0.05)	0.09	0.5 (0.0, 3.6)
		Santa Ana	206	4.7 (3.9, 6.3)	0.14	9.8 (9.6, 9.9)	**<0.001**	0.04 (0.02, 0.18)	0.31	**3.1 (1.0, 10.6)**
		Hermosillo	220	5.6 (3.9, 9.2)	0.73	9.1 (8.9, 9.2)	**<0.001**	0.15 (0.03, 0.54)	0.68	**6.7 (2.6, 20.6)**
		Obregon	342	5.3 (4.0, 8.0)	0.73	9.2 (9.1, 9.4)	**<0.001**	0.08 (0.03, 0.36)	0.97	**9.9 (3.9, 30.4)**
**2015**	Aug.	Nogales, SN	163	6.3 (4.3, 10.6)	Ref.	12.7 (12.6, 12.8)	Ref.	0.04 (0.00, 0.34)	0.37	Ref.
		Tucson	390	7.3 (4.9, 11.2)	0.1	9.1 (8.8, 9.3)	**<0.001**	0.36 (0.10, 0.67)	1.46	**3.8 (2.1, 7.0)**
		Nogales, AZ	85	6.1 (4.1, 7.8)	0.56	12.7 (12.5, 12.8)	0.43	0.04 (0.00, 0.11)	0.19	0.5 (0.1, 1.6)
		Santa Ana	201	5.2 (4.4, 8.3)	0.37	10.7 (10.1, 11.1)	**<0.001**	0.09 (0.04, 0.36)	0.54	1.4 (0.7, 2.9)
		Hermosillo	140	7.2 (5.0, 11.9)	0.14	9.9 (9.6, 10.0)	**<0.001**	0.26 (0.06, 0.65)	0.48	1.2 (0.6, 2.3)
		Obregon	174	5.1 (4.2, 7.6)	0.1	9.7 (9.5, 9.8)	**<0.001**	0.08 (0.03, 0.30)	0.43	1.1 (0.5, 2.2)
**2015**	Sept/ Oct	Nogales, SN	188	7.1 (4.8, 11.4)	Ref.	19.9 (19.4, 20.5)	Ref.	0.01 (0.00, 0.10)	0.16	Ref.
		Tucson	640	5.5 (4.1, 8.4)	**0.01**	10.8 (10.5, 11.2)	**<0.001**	0.08 (0.02, 0.31)	1.3	**7.4 (3.6, 16.3)**
		Nogales, AZ	62	4.1 (3.7, 4.5)	**<0.001**	20.7 (20.3, 21.2)	**<0.001**	0.00 (0.00, 0.00)	0.03	0.3 (0.0, 1.6)
		Santa Ana	93	5.6 (3.9, 9.6)	0.17	12.8 (12.0, 14.7)	**<0.001**	0.02 (0.01, 0.34)	0.2	1.1 (0.4, 3.1)
		Hermosillo	109	4.6 (3.9, 7.4)	**<0.001**	12.0 (11.7, 12.1)	**<0.001**	0.03 (0.00, 0.19)	0.13	0.9 (0.3, 2.4)
		Obregon	158	5.6 (4.4, 10.0)	0.22	10.8 (10.3, 11.1)	**<0.001**	0.10 (0.02, 0.45)	0.43	**2.5 (1.1, 5.9)**
**2015**		Nogales, SN	417	6.4 (4.4, 10.5)	Ref.	12.9 (12.6, 19.6)	Ref.	0.02 (0.00, 0.19)	0.2	Ref.
		Tucson	1,344	5.9 (4.1, 9.7)	0.15	9.7 (9.3, 10.6)	**<0.001**	0.13 (0.03, 0.49)	1.1	**5.1 (3.3, 8.0)**
		Nogales, AZ	220	4.6 (3.8, 6.8)	**<0.001**	12.7 (12.1, 20.1)	0.1	0.00 (0.00, 0.05)	0.1	0.4 (0.2, 1.1)
		Santa Ana	500	5.0 (4.2, 7.7)	**0.01**	10.1 (9.8, 11.1)	**<0.001**	0.06 (0.02, 0.24)	0.4	1.5 (0.9, 2.6)
		Hermosillo	469	5.8 (4.2, 9.7)	**0.34**	9.7 (9.1, 10.7)	**<0.001**	0.13 (0.02, 0.50)	0.4	**1.9 (1.2, 3.1)**
		Obregon	674	5.3 (4.2, 8.4)	**0.01**	9.6 (9.3, 9.9)	**<0.001**	0.08 (0.03, 0.36)	0.6	**2.7 (1.7, 4.3)**

[a] Logistic regression for binomial proportions [b] Wilcoxon rank sum test with continuity correction [c] Negative binomial regression model stratified by collection cycle and year, relating count of potential vectors per household collection site with log (trap days) as offset and with city as a predictor. * Ref. indicates the city used for comparisons. July Tucson 2013 missing due to differing RNA extraction methods used for mosquito samples during that collection period.
